# Association of acanthosis nigricans with metabolic syndrome – An analytic cross-sectional study^⋆^

**DOI:** 10.1016/j.abd.2022.07.006

**Published:** 2023-03-22

**Authors:** Sanjiv Choudhary, Ankita Srivastava, Vikrant Saoji, Adarshlata Singh, Isha Verma, Shivani Dhande

**Affiliations:** a Department of Dermatology, All India Institute of Medical Sciences, Nagpur, Maharashtra, India; b Department of Dermatology, Jawahar Lal Nehru Medical College, Sawangi, Wardha, Maharashtra, India

**Keywords:** Acanthosis nigricans, Association, Cross-sectional studies, Metabolic syndrome

## Abstract

**Background:**

Globally, few studies have been undertaken to assess the association of acanthosis nigricans (AN) with metabolic syndrome (MS). Most of the available studies have either focused on a particular age group, gender, ethnicity or on a single component of MS.

**Objectives:**

To determine the association between AN and MS as a whole and with all individual components of MS in adult patients of either gender.

**Material and methods:**

This was a cross-sectional study with a comparative group. Eighty-one subjects were recruited in each group. Fasting plasma glucose (FPG) and lipid profile were done. MS was defined by using the international diabetic federation (IDF) criteria. Association of body mass index (BMI), waist circumference, blood pressure, FPG, high-density lipoprotein (HDL) and triglycerides (TG) with AN was assessed by Pearson’s chi-square test followed by univariate and multivariate analysis.

**Results:**

The prevalence of MS was found to be significantly higher in the group with AN. On univariate analysis, a significant association of AN was found with BMI, waist circumference, high systolic and diastolic blood pressure, HDL, and TG. Multivariate analysis revealed a significant association between waist circumference, high systolic and diastolic blood pressure, and high TG levels with AN. The risk of MS was found to be eight times higher in cases of AN.

**Study limitations:**

The small sample size and single-center data are the limitations of the present study.

**Conclusion:**

AN is strongly associated with MS as a whole and with its individual components including increased waist circumference, hypertension, and dyslipidemia.

## Introduction

Acanthosis nigricans (AN) is a dermatosis characterized by velvety, papillomatous, brownish-black plaques, typically on the intertriginous surfaces and neck.^1^ It may also affect the eyelids, lips, vulva, mucosal surfaces, and the backs of hands, groin, knees and elbows.

Metabolic syndrome (MS) is a clustering of risk factors that are of metabolic origin and are accompanied by an increased risk of cardiovascular disease and diabetes mellitus. These risk factors include central obesity, atherogenic dyslipidemia, elevated blood pressure, and raised plasma glucose.^2^

Previous studies on the association of AN with MS are limited to either male or female gender and in the majority of those studies all the parameters defining MS had not been taken into account. The study carried out by Ayaz et al. included only obese and overweight female subjects;^3^ while another study from India was carried out on young males.^4^ Two more studies from India had focused on the association of AN only with insulin resistance and glucose metabolism but had not correlated AN with other defining criteria of MS like hypertension and dyslipidemia.^5^, ^6^ Therefore, limited information focusing on the association between AN and MS is available at present. Hence, this study was undertaken with the aim to study the association of AN with all individual components of MS and MS as a whole amongst adult patients of both genders.

## Materials and methods

This cross-sectional study with a comparative group was conducted at a tertiary care center after taking clearance from the institutional ethics committee. Patients were divided into a study and comparative group consisting of 81 patients each (n = 162). Patients were recruited from South Asian region (Indian subcontinent). The study group consisted of cases with AN and the comparative group consisted of age and sex-matched subjects without AN. The sample size was calculated by assuming the prevalence of MS in patients with and without AN to be 60% and 30% respectively. Considering the confidence level at 95% and allowable error to be 10%‒20% of prevalence, the sample size was calculated to be 67 in each group.

The inclusion criteria for both groups were subjects ages between 20 to 55 years of both genders. Exclusion criteria were subjects on drugs that are likely to induce AN, patients of syndromic (childhood-onset) AN, and patients with a history of smoking and alcohol intake.

Detailed history taking and clinical examination were done in all the subjects. The patients were examined to note the location and morphology of skin lesions. Anthropometric and clinical measurements like weight, height, BMI, waist circumference, and blood pressure were recorded. Relevant blood investigations including fasting plasma glucose and lipid profile were done. The international diabetic federation (IDF) criteria were used to define MS (Table 1).^7^ The association between each individual parameter and AN were tested for statistical significance using Pearson’s Chi-square test. Univariate analysis was performed to find out the association of variables with AN by calculating the odds ratio. Subsequently, multivariate analysis was performed by including variables that were found to have a statistically significant effect in univariate analysis. All the analyses were performed using SPSS version 20.0 (IBM Corp.) software. The statistical significance was tested at a 5% level.

**Table 1:** International diabetic federation criteria for metabolic syndrome (MS)*.

According to the IDF definition, for a person to be defined as having the metabolic syndrome they must have: Central obesity (defined as waist circumference with ethnicity specific values) plus any two out of the four factors described below
1) Elevated waist circumference: Population and country-specific definitions (South Asians Based on a Chinese, Malay, and Asian-Indian population: Males ≥90; Females ≥80 cm) plus two out of following four factors:
2) Elevated triglycerides (drug treatment for elevated triglycerides is an alternate indicator) ‒ ≥ 150 mg/dL (1.7 mmoL/L) or history of specific treatment for lipid abnormality
3) Reduced HDL-C (drug treatment for reduced HDL-C is an alternate indicator): < 40 mg/dL (1.0 mmoL/L) in males; < 50 mg/dL (1.3 mmoL/L) in Females or specific treatment for this lipid abnormality
4) Elevated blood pressure (antihypertensive drug treatment in a patient with a history of hypertension is an alternate indicator): Systolic ≥ 130 and/or diastolic ≥85 mm/Hg or on treatment for previously diagnosed hypertension
5) Elevated fasting glucose (drug treatment of elevated glucose is an alternate indicator): ≥ 100 mg/dL or previously diagnosed type 2 diabetes mellitus

* Source: Alberti KG, et al. 2006.^7^

## Observation and results

In this study, the subjects were equally distributed in terms of age group and gender in both AN and comparative groups with no statistical difference (p = 0.14 and 0.75 respectively). The age range was 20 to 55 years with a mean of 32.82 ± 10.19 years for the AN group and 33.67 ± 8.09 years for comparative subjects. The difference in BMI for cases and comparative subjects was statistically significant (p = 0.0001). The significant association between BMI and AN occurrence was due to a higher proportion of obese I & obese II subjects in cases with AN as compared to comparative subjects without AN. The proportion of subjects with abnormally high waist circumference was significantly higher in AN cases as compared to subjects in comparative group for both males and females (p < 0.0001). Both high systolic and high diastolic blood pressure was observed in AN cases as compared to comparative subjects, which was statistically significant (p = 0.0002). No statistically significant difference was found in fasting plasma glucose (FPG) levels in both groups (p = 0.32). A statistically significant difference was observed in HDL and triglyceride (TG) levels in the AN cases as compared to the comparative group (Table 2).

**Table 2:** Distribution of subjects according to BMI, waist circumference, systolic blood pressure (SBP) and diastolic blood pressure (DBP), fasting plasma glucose (FPG), high density lipoprotein (HDL) and triglycerides (TG) levels in acanthosis nigricans (AN) cases and comparative groups.

Parameter value studied	Number [out of 81] in AN case group (%)	Number [out of 81] in comparative group (%)	Total [out of 162]	χ^2^ Statistic	p-value
**BMI (kg/m^2^)**					
Normal (18.5‒22.9)	16 (19.7)	55 (67.9)	71 (43.8)	41.004	<0.0001
Pre-obese (23‒24.9)	10 (12.3)	8 (9.9)	18 (11.1)		
Obese I (25‒29.9)	30 (37.0)	12 (14.8)	42 (25.9)		
Obese II (30 & above)	25 (30.8)	6 (7.4)	31 (19.1)		
Total	81	81	162		
**Waist circumference (cm)**					
Males					
Normal (< 90)	7 (8.6)	37 (45.7)	44 (27.2)	41.76	<0.0001
Abnormal (≥ 90)	40 (49.4)	8 (9.8)	48 (29.6)		
Females					
Normal (< 80)	4 (4.9)	27 (33.3)	31 (19.1)	28.34	
Abnormal (≥ 80)	30 (37.1)	9 (11.1)	39 (24.1)		
Total	81	81	162		
**Blood pressure (mmHg)**					
SBP < 130 &	53 (65.4)	58 (71.6)	111 (68.5)	21.27	<0.0001
DBP < 85					
SBP ≥ 130 &	8 (9.9)	21 (25.9)	29 (17.9)		
DBP < 85					
SBP < 130	3 (3.7)	1 (1.2)	4 (2.5)		
& DBP ≥ 85					
SBP ≥ 130	17 (20.9)	1 (1.2)	18 (11.1)		
& DBP ≥ 85					
Total	81	81	162		
**FPG (mg/dL)**					
Normal (FPG < 100)	62 (76.5)	67 (82.7)	129 (79.6)	0.9514	0.3293
Abnormal (FPG ≥ 100)	19 (23.4)	14 (17.2)	33 (20.4)		
Total	81	81	162		
**HDL (mg/dL)**					
Normal (≥ 40)	18 (22.2)	39 (48.1)	57 (35.2)	11.936	0.0005
Abnormal (< 40)	63 (77.8)	42 (51.9)	105 (64.8)		
Total	81	81	162		
**TG (mg/dL)**					
Normal (TG < 150)	45 (55.5)	69 (85.2)	114 (70.3)	17.053	<0.0001
Abnormal (TG ≥ 150)	36 (44.4)	12 (14.8)	48	(29.7)	
Total	81	81	162		

MS was present in 46 (56.8%) out of 81 AN cases and 11 (13.6%) out of 81 comparative subjects, this difference in proportion was statistically significant (p < 0.0001) (Table 3).

**Table 3:** Distribution of subjects according to presence or absence of MS in AN cases and comparative groups.

	AN Case group	Comparative group	Total		
Metabolic syndrome	No. (%)	No. (%)	No. (%)	χ^2^ Statistic	p-value
Absent	35 (43.2)	70 (86.4)	105 (64.8)		
Present	46 (56.8)	11 (13.6)	57 (35.2)	33.1579	<0.0001
Total	81	81	162		

Univariate analysis (Table 4) depicted a significant association of AN with BMI, obese I, obese II, waist circumference, high systolic and diastolic blood pressure, HDL, TG and MS. FPG, and only high diastolic blood pressure showed a greater risk of association in cases but it was statistically insignificant. Only high systolic blood pressure showed no risk of association in cases with AN with statistically insignificant p-value.

**Table 4:** Univariate and multivariate analysis for the association of demographic and various metabolic factors in subjects with and without AN.

Parameters with cut-off value	Unadjusted OR (95% CI)	p-value	Adjusted OR^a^ (95% CI)	p-value
Gender				
Male		1.00		1.00
Female	0.91 (0.48‒1.69)	0.751	0.79 (0.29‒2.09)	0.634
BMI (kg/m^2^)				
Normal (18.5‒22.9)		1.00		
Pre-obese (23‒24.9)	4.19 (1.41‒12.98)	0.0059		
Obese I (25‒29.9)	8.33 (3.56‒20.76)	<0.0001		
Obese II (30 & above)	13.63 (5.00‒42.89)	<0.0001		
Waist Circumference (cm)				
Normal		1.00		1.00
Abnormal	23.9 (10.44‒54.98)	<0.0001	15.78 (6.16‒40.45)	<0.0001
BP (mmHg)				
Systolic < 130 & Diastolic < 85		1.00		1.00
Systolic ≥ 130 & Diastolic < 85	0.42 (0.16‒1.01)	0.0512	0.36 (0.11‒1.25)	0.108
Systolic < 130 & Diastolic ≥ 85	2.99 (0.34‒87.81)	0.2841	4.08 (0.19‒87.64)	0.369
Systolic ≥ 130 & Diastolic ≥ 85	16.2 (3.15‒398.8)	0.0002	12.98 (1.24‒135.7)	0.032
FPG (mg/dL)				
Normal (FBS < 100)		1.00		
Abnormal (FBS ≥ 100)	1.46 (0.67‒3.22)	0.3293		
HDL (mg/dL)				
Normal		1.00		1.00
Abnormal	3.21 (1.64‒6.49)	0.0005	1.69 (0.58‒4.95)	0.334
TG (mg/dL)				
Normal (TG < 150)		1.00		1.00
Abnormal (TG ≥ 150)	4.52 (2.17‒9.99)	<0.0001	2.97 (1.04‒8.48)	0.042
Metabolic syndrome				
Absent		1.00		
Present	8.17 (3.87‒18.53)	<0.0001		

a Multivariate logistic regression analysis: HL = 0.475.

Table 4, Table 5 showed multivariate analysis for the association of demographic and various metabolic factors in subjects with and without AN. The multivariate analysis was performed in two parts. In the first part (Table 4), demographic variables along with statistically significant predictors of MS (Waist circumference, BP, HDL, TG) were included in the model. BMI and waist circumference indicated a strong positive correlation resulting in a co-linearity effect. Hence, BMI was excluded, and the analysis was performed retaining waist circumference in the model. The Hosmer-Lemeshow test resulted in a p-value of 0.475 indicating a reasonably good model fit.

**Table 5:** Multivariate analysis for the association of gender and MS in subjects with and without AN.

Parameter	Levels	Unadjusted OR (95% CI)	p-value	Adjusted OR^a^ (95% CI)	p-value
Gender	Male		1.00		1.00
	Female	0.91 (0.48‒1.69)	0.751	0.69 (0.34‒1.45)	0.338
Metabolic	Absent	1.00		1.00	
Syndrome	Present	8.17 (3.87‒18.53)	<0.0001	7.67 (3.43‒17.15)	<0.0001

a Multivariate logistic regression analysis: HL test = 0.995.

The effect of gender was nearly unchanged in the multivariate analysis (Table 5), while abnormal waist circumference continued showing high odds, indicating a significant association with AN. Further, abnormally high systolic and diastolic blood pressure had an associated OR of 12.98 which proved its significant association with AN. Abnormally high TG levels also showed a significant association with AN. MS too indicated increased odds with p-value < 0.0001. The model fitness was good as indicated by Hosmer-Lemeshow test with p-value of 0.995.

## Discussion

AN has been reported to be associated with MS or syndrome X. However, many previously available reports are focused on a particular age group, gender, ethnicity, or a particular component of the syndrome, such as obesity and insulin resistance.^3^, ^4^, ^5^, ^6^ The present study was carried out on adult Indian subjects, including both males and females.

Observations from various past studies^3^, ^4^, ^8^ have indicated that AN is associated with high BMI and waist circumference. The present study also shows similar findings. However, no association was observed between the grading of AN and BMI in a cross-sectional study by Venkatswami et al.^5^

Obesity-associated AN is the most common type of AN. The dermatosis is weight dependent, and lesions may completely regress with weight reduction. The pathogenesis of AN with obesity can be explained on the basis of secondary insulin resistance, where the target cells fail to respond to normal levels of circulating insulin, resulting in compensatory hyperinsulinemia. Hyperinsulinemia elevates serum concentrations of free insulin-like growth factor-1 (IGF-1) and androgens, while simultaneously reducing insulin-like growth factor-binding protein 3 (IGFBP-3) and sex hormone-binding globulin. Free IGF-1 is a potent mitogen for virtually all tissues. Excessive concentrations of insulin and free IGF-1 bind to IGF-1 receptors (IGF-R) on keratinocytes and dermal fibroblasts, thereby resulting in the development of AN.^9^ Besides, excess abdominal adipose tissue in obese individuals also results in the release of increased amounts of free fatty acids which directly affect insulin signaling, diminish glucose uptake in muscle, drive exaggerated triglyceride synthesis and induce gluconeogenesis in the liver.^10^

High blood pressure is among the criteria for establishing the diagnosis of MS. In the present study, the proportion of subjects with both systolic and diastolic BP above normal levels was significantly higher in the AN group as compared to the comparative group. This is in accordance with a previous study.^8^ In some other studies, AN was found to be significantly associated with either high systolic or diastolic blood pressure.^4^ The relationship between AN and hypertension can be explained on the basis of insulin resistance as the common pathomechanism that is known to contribute to both conditions. In the setting of insulin resistance, the vasodilatory effect of insulin is lost but the effect on renal sodium reabsorption is preserved; thereby causing an increase in blood pressure. Hyperinsulinemia also leads to increased sympathetic nervous system activity which also contribute to hypertension.^10^ On the other hand, Venkatswami et al.^5^ observed no association between AN grading and hypertension.

Hyperglycemia is another component of MS. In our study, however, we did not find a statistically significant difference between FPG levels of AN cases and comparative groups. In some of the previous studies^3^, ^5^, ^8^ conducted in India and other countries, a significant association of AN cases with fasting insulin and HOMA-IR was noted, but no association with FPG could be established. This could be explained by the fact that compensatory hyperinsulinemia (due to insulin resistance) in such cases is often able to maintain glucose homeostasis in the initial stages; thus, the blood glucose in these patients generally remains in the normal range and this indicates a prediabetic condition. On the contrary, another study^6^ demonstrated a statistically significant correlation of AN with fasting glucose in addition to fasting insulin and HOMA-IR.

Dyslipidemia in MS is characterized by deranged HDL and/or TG. In the present study, derangement of both HDL and TG was found to be significantly associated with AN in univariate analysis. This is in concordance with the studies conducted by Ayaz et al.^3^ and Jorwal et al.^4^ These abnormalities in HDL and/or TG levels can be explained by central obesity and compensatory hyperglycemia (secondary to insulin resistance). On the other hand, some other studies^5^, ^8^ revealed no association of AN with HDL and TG.

There is a paucity of studies regarding the association of AN with MS. Few past studies have demonstrated the association of AN with MS.^3^ Kamel et al.^8^ in their study also concluded that the severity of AN correlates positively with biochemical changes related to the MS. Majority of the previous studies focused on the association of AN with individual components of MS and not MS as a whole (at least three components). In the present study, we evaluated both groups to determine the prevalence of MS. We used the IDF criteria to establish the diagnosis of MS. MS was present in 56.8% of AN cases versus 13.6% in the comparative group, and the difference of proportion was statistically highly significant.

In the present study majority of cases with AN (86.5%) were obese and obesity is one of the major causes of insulin resistance. Insulin resistance is one of the main links in the association between obesity-induced AN and MS as well as its individual components.

Univariate analysis in our study depicted a significant association of AN with BMI, waist circumference, high systolic and diastolic blood pressure, HDL, TG and MS. This is in concordance with a previous case–control study^11^ where univariate analysis showed a statistically significant association of high BMI, abnormal waist circumference, diastolic BP and MS with AN. Additionally, a significant association of AN noted with fasting blood sugar, while no association of AN with abnormal HDL was noted. Univariate analysis of TG was not done in this study.^11^

Our study, on multivariate analysis, demonstrated a significant association between abnormal waist circumference, high systolic and diastolic blood pressure, and high TG levels with AN. In the previous study by Acharya et al.,^11^ multivariate analysis showed a significant association of abnormal waist circumference and MS with AN, while no such association was noted with abnormal HDL level.

Small sample size is the major limitation of this study. Multicentric studies with a larger number of subjects are needed to substantiate the results of the present study.

## Conclusion

AN is strongly associated with individual components of MS-like high waist circumference, high systolic & diastolic blood pressure, and high TG level. It also shows a strong association with MS as a whole (abnormal waist circumference with any two parameters defining MS). Patients of AN have an eight times greater risk of MS as compared to subjects without AN.

## Financial support

None declared.

## Authors' contributions

Sanjiv Choudhary: The study concept and design; data collection, or analysis and interpretation of data; statistical analysis; writing of the manuscript or critical review of important intellectual content; effective participation in the research guidance; intellectual participation in the propaedeutic and/or therapeutic conduct of the studied cases; critical review of the literature; final approval of the final version of the manuscript.

Ankita Srivastava: Analysis and interpretation of data; statistical analysis; writing of the manuscript or critical review of important intellectual content, critical review of the literature; final approval of the final version of the manuscript.

Vikrant Saoji: The study concept and design; data collection, or analysis and interpretation of data; statistical analysis; critical review of important intellectual content; effective participation in the research guidance; critical review of the literature; final approval of the final version of the manuscript.

Adarshlata L. Singh: The study concept and design; data collection, or analysis and interpretation of data; statistical analysis; final approval of the final version of the manuscript.

Isha Verma: Data collection, or analysis and interpretation of data; writing of the manuscript or effective participation in the research guidance; final approval of the final version of the manuscript.

Shivani Dhande: Data collection, or analysis and interpretation of data; writing of the manuscript or effective participation in the research guidance; final approval of the final version of the manuscript.

## Conflicts of interest

None declared.
